# Chemical Synthesis of Oligosaccharides Derived from Serotype 35B and D Provides Molecular Insight in l‑Ficolin Binding

**DOI:** 10.1021/jacs.5c12005

**Published:** 2025-08-06

**Authors:** Ivan A. Gagarinov, Lin Liu, Francesco Torricella, John N. Glushka, Alpeshkumar K. Malde, Mark von Itzstein, Geert-Jan Boons

**Affiliations:** † Chemical Biology and Drug Discovery, Utrecht Institute for Pharmaceutical Sciences, and Bijvoet Center for Biomolecular Research, 1355 Utrecht University, Utrecht 3584 CG, the Netherlands; ‡ Complex Carbohydrate Research Center, University of Georgia, Athens, Georgia 30602, United States; § Department of Chemistry, University of Georgia, Athens, Georgia 30602, United States; ∥ Laboratory of Chemical Physics, 2511 National Institute of Diabetes and Digestive and Kidney Diseases, National Institutes of Health, Bethesda, Maryland 20892-0520, United States; ⊥ Institute for Biomedicine and Glycomics, Gold Coast Campus, Griffith University, Southport QLD 4222, Australia

## Abstract

The wide use of capsular polysaccharide (CPS) conjugate vaccines is causing serotype replacement, and the emergence of serotype 35B is concerning because of multidrug resistance. CPS of 35B is composed of pentasaccharide repeating units that are linked through phosphodiester linkages. One of the galactofuranose residues of the pentasaccharide is acetylated, which distinguishes it from invasive serotype 35D, lacking the acetyl ester. Here, we describe a synthetic approach that can provide oligosaccharides derived of CPS 35B and 35D composed of up to 15 monosaccharides using a pentasaccharide building block equipped with four orthogonal protecting groups. The synthetic compounds were used to examine binding properties of l-ficolin, which is a protein that can activate the lectin pathway of the complement system. Solution-phase NMR experiments and computational modeling demonstrate that Galf­(OAc)-1,1-Ribitol of the repeating unit of 35B CPS constitutes the minimal motif for binding to l-ficolin, and the acetyl ester is a key recognition motif. Microarray binding experiments confirmed that *O*-acetylation is essential for recognition and that oligosaccharides composed of 2 or 3 repeating units bind avidly due to ficolin’s multimeric structures. The data provide a rationale why 35D may escape immune detection and be more invasive. The oligosaccharides were employed to investigate binding to pneumococcal serum factors 35a and 29b, which indicates that immunization with 35B CPS will not provide protection against 35D. Antibodies that can bind 35D can, however, recognize 35B, and thus, 35D CPS may provide cross-protection.

## Graphical abstract

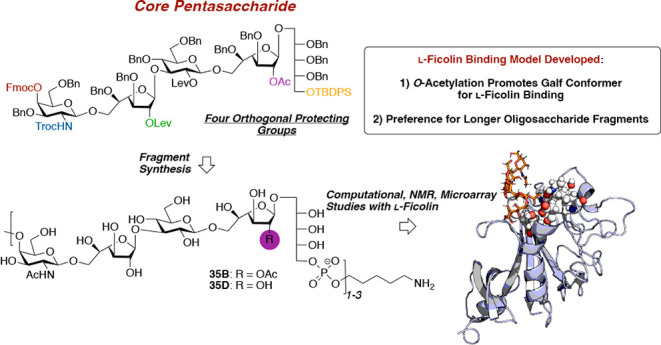

## Introduction

 are gram-positive bacteria that are a common cause of pneumonia and can trigger other illnesses, including severe ear infections, meningitis, and bacteremia.^1^ It can result in long-lasting problems, such as hearing loss, brain damage, and even death. The most effective way to combat pneumococcal disease is by preventative vaccination. More than 90 serologically distinct pneumococcal capsular polysaccharides (serotypes) have been identified. Antibodies against the capsule are protective, and therefore, polysaccharide conjugate vaccines have been developed against the most prevalent serotypes, which have resulted in a great reduction in pneumococcal disease.^2^ The most widely employed vaccine, which is known as PCV13, is composed of capsular polysaccharides (CPSs) conjugated to carrier proteins and provides protection against 13 serotypes that commonly cause pneumococcal disease. The wide use of PCV13, and the earlier PCV7 vaccine, has caused other serotypes to become more prevalent.^3^ This so-called antigenic shift is favoring the spread of invasive nonvaccine serotypes, such as 12F, 15B, 15C (15B/C), and 35B/D. Serotype 35B is of particular concern because it is associated with high rates of multidrug resistance.^4–6^ The emergence of new serotypes has led to the recent introduction of 20 and 21 valent vaccines, in which the capsular polysaccharides are conjugated to the carrier protein CRM_197_.

CPS of 35B is a high molecular weight polymer composed of d-galactofuranose, d-glucose, *N*-acetyl-d-galactosamine, and ribitol (Figure 1).^7^ These monosaccharides are *trans*-linked through glycosidic linkages to give a pentasaccharide repeating unit that is further polymerized through phosphodiesters. One of the galactofuranose residues is modified at C-2 by an acetyl ester. This feature distinguishes 35B from the closely related and invasive serotype 35D that lacks the acetyl ester.^8^

**Figure 1:**
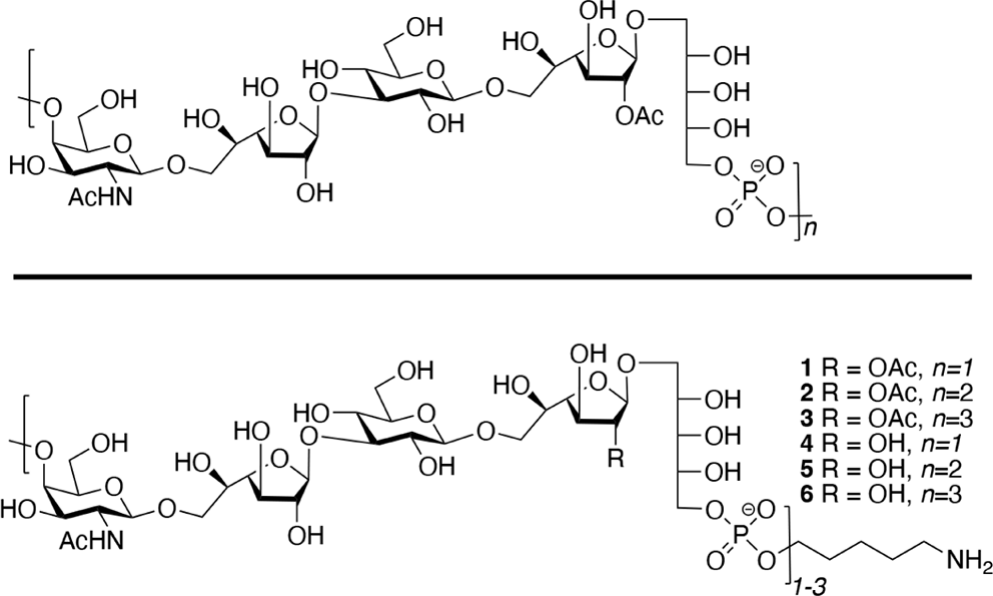
Structure of 35B CPS and well-defined linker-equipped target oligosaccharides.

*O*-acetylation is important for functional immunity for a number of polysaccharides, such as meningococcal serogroup A^9^ and V.^10^ Furthermore, *O*-acetylation can be important for innate immune detection, and for example, *O*-acetylated capsular polysaccharides can be recognized by the serum protein l-ficolin, which is an initiator of the lectin complement pathway.^11^ l-Ficolin-mediated complement deposition has been observed for serotypes 11A and 35B but not for *O*-acetyltransferase-deficient derivatives of these serotypes. The ability of l-ficolin to recognize serotype 11A and 35B capsular polysaccharides has been associated with low invasiveness in children.^12^ The elderly are, however, susceptible to invasive pneumococcal diseases by these serotypes, and it has been proposed that this is due to reduced l-ficolin-mediated immunity. Thus, the elderly are an important focus group for vaccination by serotype 35B. Furthermore, serotype 35D, which lacks the C-2 acetyl ester at the galactofuranoside, is more invasive^13,14^ which may be due to a lack of recognition by l-ficolin.

Ficolins 1, 2, and 3, which are also known as ficolin-m, ficolin-l, and ficolin-h, are human serum-associated pattern-recognition receptors that are structurally and functionally similar to mannose-binding lectin (MBL).^11^ Ficolins can form complexes with MBL-associated serine proteases and, upon binding, initiate the lectin complement pathway, resulting in opsonophagocytosis. Ficolins have a collagen-like domain that is important for oligomerization and a fibrinogen-like domain that can recognize specific carbohydrate motifs. The three human ficolins have different ligand requirements and can distinguish self from non-self. l-Ficolin can recognize a diverse range of carbohydrates, including acetylated *N*- and *O*-structures, as well as neutral sugar polysaccharides. The ligand requirements of ficolins have mainly been studied by genetic approaches, and for example, the importance of *O*-acetylation of CPS of serotypes 11A and 35B has been determined by mutants lacking *O*-acetyltransferases.^12^ A series of biologically relevant oligosaccharides is required to dissect how l-ficolin selectively recognizes microbial polysaccharides for complement-mediated neutralization.

Here, we report the chemical synthesis of well-defined oligomers derived from CPS 35B. By saponification of the acetyl esters, compounds derived from serotype 35D were easily obtained. The synthetic repeating unit of CPS 35B was employed in Rotating Frame Overhauser Effect Spectroscopy (ROESY) and Saturation Transfer Difference (STD) Nuclear Magnetic Resonance (NMR) experiments with l-ficolin. We identified acetylated Galf2 and ribitol as the key monosaccharide units of the repeating unit involved in l-ficolin binding, which was supported by computational docking. Microarray binding experiments demonstrated that *O*-acetylation is essential for recognition of CPS 35B by l-ficolin and showed that oligosaccharides having 2–3 repeating units bind more avidly likely due to l-ficolin’s oligomeric structure. Collectively, the binding data provide a rationale why the closely related serotype 35D might escape immune detection and be more invasive.^13^ The synthetic oligosaccharides were also investigated for binding to pneumococcal serum factors 35a and 29b, which are employed for serotype identification. Factor serum 35a is reported to bind only serotype 35B, and as expected, it only recognized *O*-acetylated oligosaccharides in a length-dependent manner. Factor serum 29b is reported to recognize both 35B and 35D and binds to all synthetic compounds. The findings have direct implementation for the development of the next-generation pneumococcal vaccine and provide an understanding of disease severity by the emerging serotypes 35B and 35D.

## Results and Discussion

### Chemical Synthesis of 35B Oligosaccharides

The chemical synthesis of the pentasaccharide repeating unit of 35B and its oligomers has not been reported yet. We envisaged a scalable synthetic strategy that can provide oligosaccharides **1**–**3** (Figure 1) that are composed of one, two, and three repeating units of the CPS of 35B. The compounds are equipped with an artificial aminopentyl linker to facilitate bioconjugation and microarray printing. Our strategy employs glycosyl acceptor **7** and thioglycosyl donors **8**–**11** to assemble key oligosaccharide **12** (Figure 2). The latter compound is equipped with four orthogonal protecting groups, *t-*butyldiphenylsilyl (TBDPS), fluorenylmethyl (Fmoc), levulinoyl (Lev), and 2,2,2-trichloroethylcarbonyl (NHTroc), which allow for oligomer assembly and deprotection without affecting the critical acetyl esters. The TBDPS and Fmoc protecting groups of **12** are at sites where a phosphodiester needs to be installed and can independently be cleaved by HF-pyridine and triethylamine, respectively.^15^ Removal of the TBDPS ether of **12** will give an alcohol that can be modified as an *H*-phosphonate to provide building block **13** that was linked to a properly protected aminopentyl spacer. Alternatively, the Fmoc protecting group of **12** can be removed by a hindered base to give an alcohol that can be coupled with *H*-phosphonate **13** to give, after oxidation with iodine, a phosphodiester. The process of Fmoc removal and coupling to **13** can be repeated to give larger oligomers.

**Figure 2:**
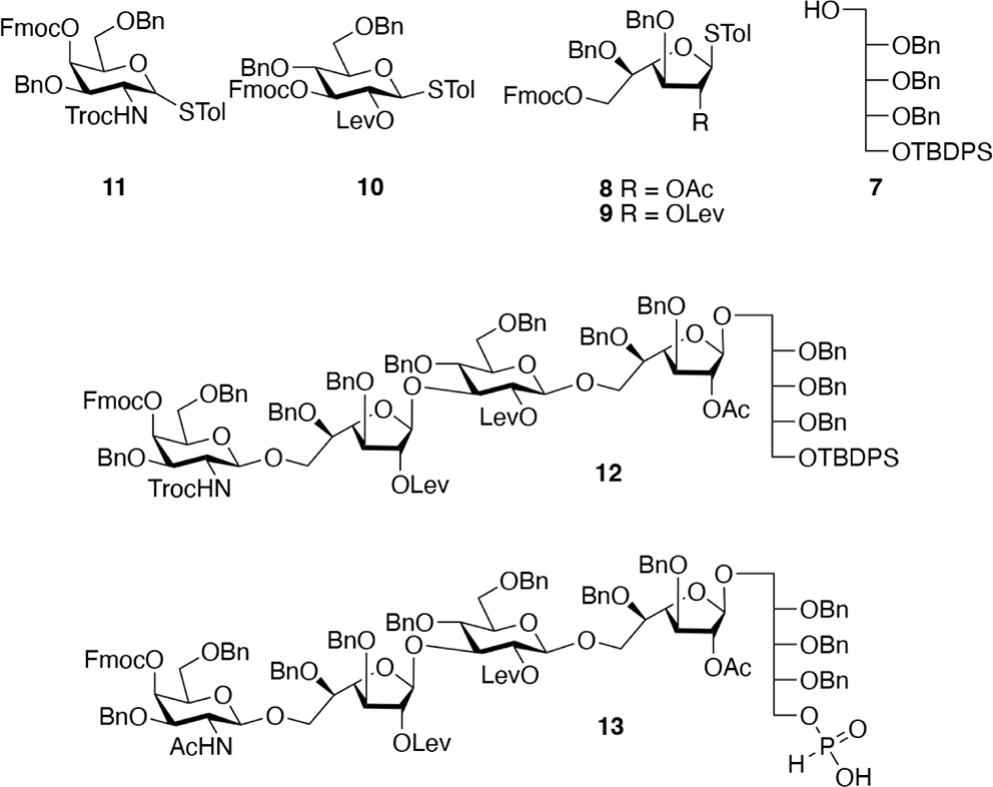
Key building blocks required for the assembly of 35B/D.

The building blocks **8**–**11** were strategically selected for efficient oligosaccharide assembly and final deprotection. The anomeric STol^16^ of donors **8**–**11** ensures shelf stability yet allows highly efficient glycosylations using *N*-iodosuccinimide (NIS)/trimethylsilyl trifluoromethanesulfonate (TMSOTf) as the promotor system.^17^ All donors are protected by Fmoc at sites where a subsequent glycosylation needs to be performed, and thus, only one set of reaction conditions is needed for glycosylation and acceptor generation, opening possibilities for automated synthesis.^16^ The Lev esters of **9** and **10** ensure that the glucoside and non-acetylated Galf moiety are selectively installed as β-anomers due to neighboring group participation.^18^ Building block **8** is used to install the Galf moiety carrying the critical acetyl ester. At the final deprotection stage, the Lev esters can be cleaved by hydrazine acetate, and these conditions preserve the base-sensitive acetyl esters. Finally, donor **11** is derivatized with an NHTroc at C2 to ensure selective β-glycoside formation, and this group can be selectively converted into a native acetamido moiety by treatment with Zn powder without affecting other parts of the oligosaccharide.

Ribitol acceptor **7** was prepared in a large quantity (∼45.0 g) in four steps from crystalline ribose dithioacetal (Scheme S1).^19^ Previous syntheses of ribitol acceptors are lengthy^20^ and not scalable. Glycosyl donors **8**–**11** could also be prepared on large scales, and details are provided in Schemes S2–S4. An NIS/TMSOTf-catalyzed^21^ glycosylation of **7** with **8** furnished disaccharide **14** in a yield of 95%, and as expected, only the β-anomer was formed due to the neighboring group participation of the acetyl ester (Scheme 1). Et_3_N-mediated cleavage of the Fmoc protecting group liberated a hydroxyl to give acceptor **15** that was coupled with the glucosyl donor **10** to provide trisaccharide **16** in a yield of 72%. Next, the Fmoc protecting group of **16** was removed using standard conditions to afford acceptor **17** that was coupled with the Galf donor **9** using NIS/TMSOTf as the promotor to provide tetrasaccharide **18** in 86% yield. The Fmoc protecting group of **18** was cleaved to give acceptor **19**, which was further coupled with glycosyl donor **11** to provide **12** in a yield of 91%. Interestingly, **12** was obtained in a much lower yield when a trichloroacetimidate^22^ donor was used. The synthetic route made it easy to obtain pentasaccharide **12** in a large quantity (15.7 g). The results demonstrate that Fmoc is an attractive temporary protecting group that can be repeatedly cleaved without affecting the other functionalities.

**Scheme 1:**
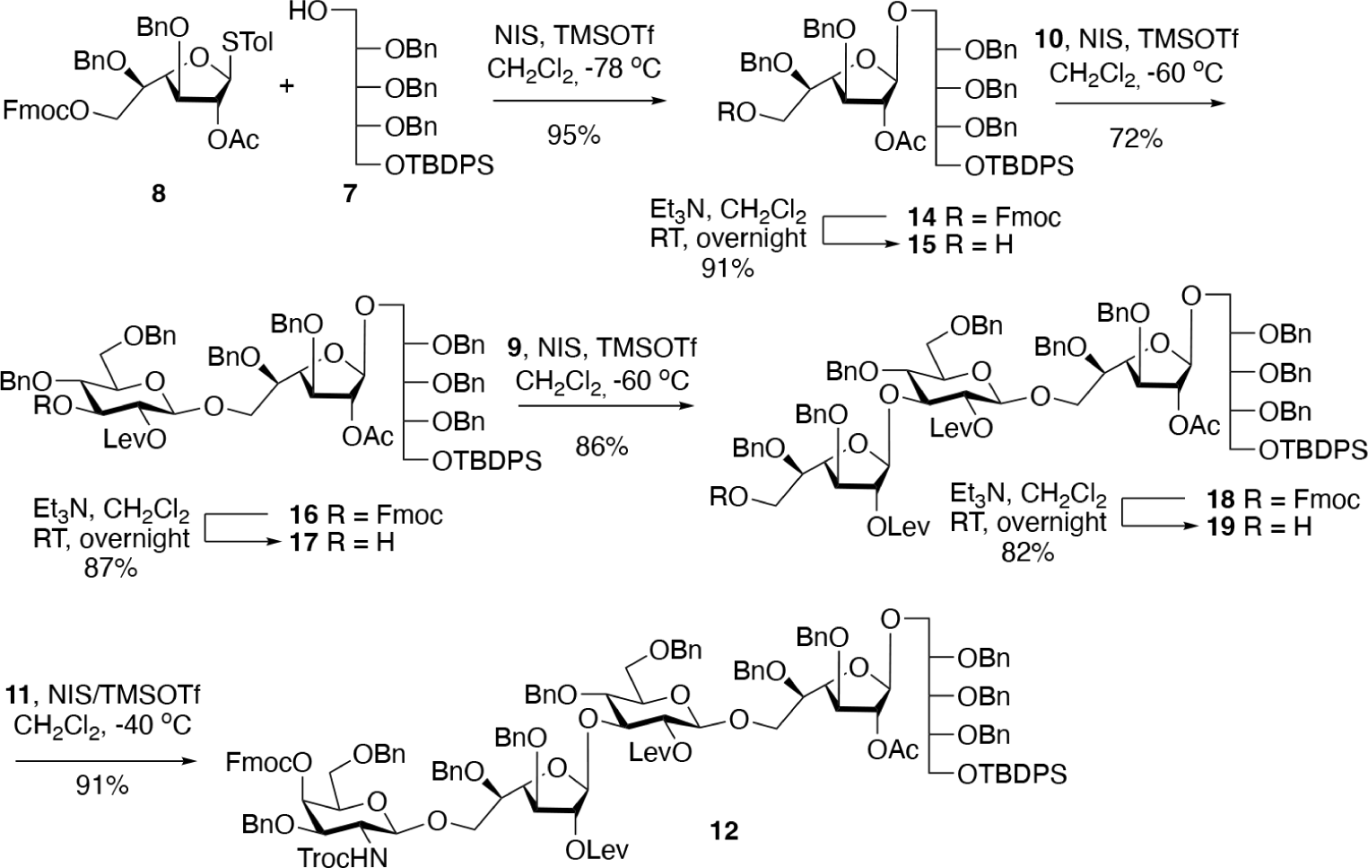
Synthesis of Core Pentasaccharide **12**

The flexibility of building block **12** was demonstrated by performing a phosphitylation followed by linker attachment and deprotection to give compound **1** (Scheme 2). Thus, the Troc carbamate of **12** was converted into an acetamido moiety by treatment with Zn dust in a solution of THF/AcOH/Ac_2_O^17^ to afford **20** in high yield. Next, the TBDPS ether of **20** was cleaved using a hydrogen fluoride–pyridine complex to provide alcohol **21** in quantitative yield. We employed commercially available salicyl chlorophosphite^23^ to install a phosphodiester between **1** and *N*-(benzyl)­benzyloxycarbonyl-protected aminopentanol.^24^ Salicyl chlorophosphite can react with alcohols to give *H*-phosphonates, which upon treatment with pivaloyl chloride (PivCl) gives a mixed phosphonic–carboxylic anhydride intermediate that in the presence of pyridine can react with an alcohol to form an *H*-phosphonate diester.^25^ Oxidation of the latter intermediate provides a phosphodiester. Thus, pentasaccharide **21** was treated with salicyl chlorophosphite^23^ to give the crucial *H*-phosphonate **13** in a yield of 89%. Coupling of the latter compound with *N*-(benzyl)­benzyloxycarbonyl-protected aminopentanol^24^ was achieved using PivCl as the activator, followed by *in situ* oxidation with iodine in pyridine/water. Subsequent removal of the Fmoc group generated fragment **22** in 88% yield over two steps.^20^ Attempts to synthesize the corresponding phosphoramidite of the pentasaccharide were unsuccessful, and the intermediate was unstable and could not be isolated. In contrast, the *H*-phosphonate route is robust, reproducible, and suitable for scale-up, making it a practical choice for our synthetic goals. Finally, the Lev esters were selectively cleaved using hydrazine acetate in a mixture of CH_2_Cl_2_ and CH_3_OH,^18^ and the remaining benzyl ethers were removed by hydrogenation over palladium hydroxide (Degussa type)^18^ at ambient pressure to furnish the first desired target compound **1**.

**Scheme 2:**
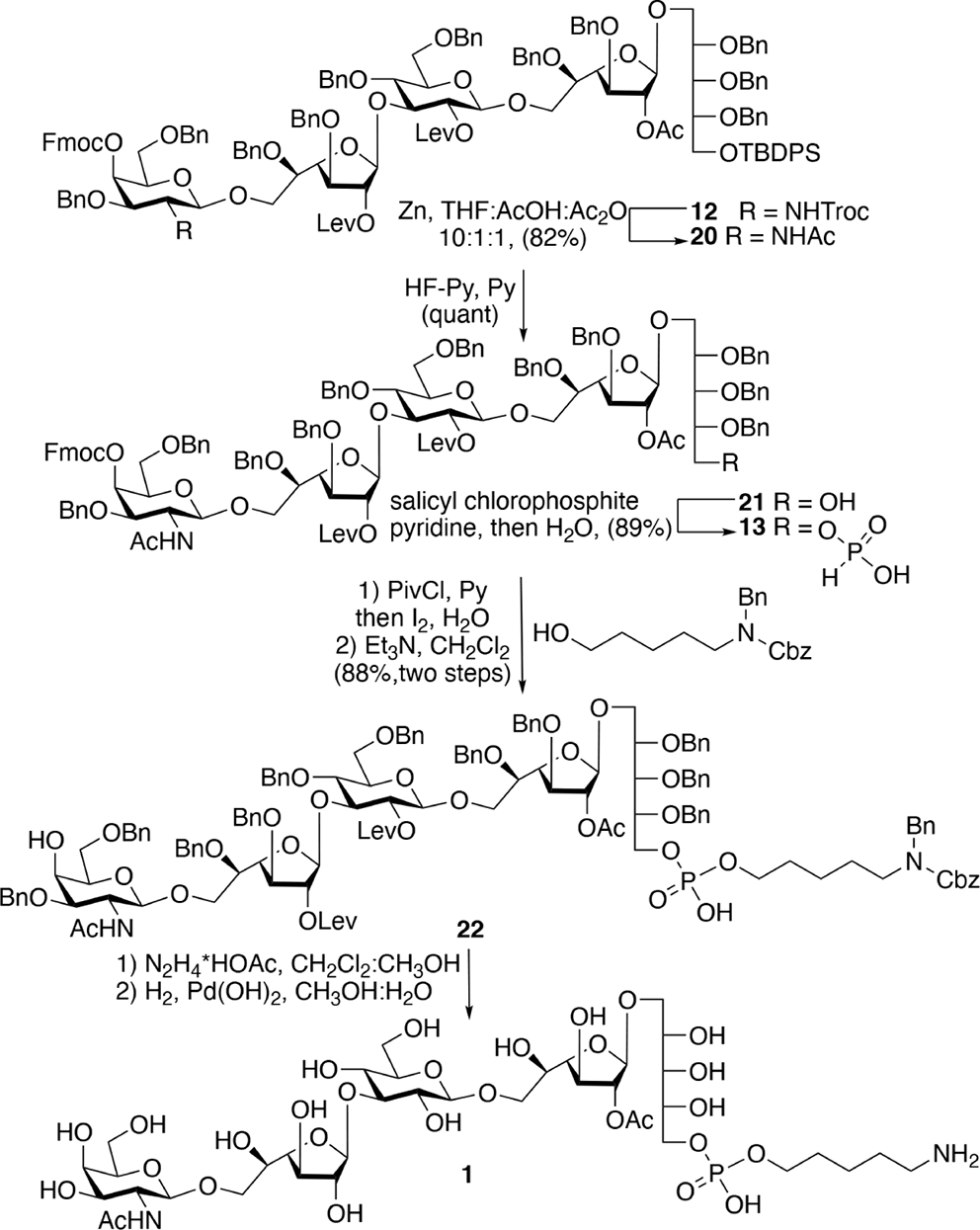
Synthesis of Key Phosphonate **13** and Its Further Functionalization with a C5 Linker to Give **1**

The versatility of phosphonate **13** was showcased by synthesizing deca- and pentadecasaccharides **2** and **3**, respectively (Scheme 3). A 5 + 5 block coupling of **22** and **13** using PivCl as an activator followed by oxidation with iodine in pyridine/water afforded **23** in a yield of 46% after purification by silica gel chromatography. The remaining starting material **22** could only be removed from the desired **23** by incorporation of acetonitrile into the eluent system, which is unusual for standard silica gel columns (see Supporting Information). Subsequent removal of the Fmoc group gave alcohol **24,** which was further treated with hydrazine acetate to remove the Lev esters and then subjected to catalytic hydrogenation over Pd­(OH)_2_ to remove all 24 benzyl ethers to provide decasaccharide **2** in an overall yield of 88%. Pentadecasaccharide **3** was synthesized according to the above methodology, albeit the coupling yield between **24** and **13** was lower (41%), which was attributed to the low nucleophilicity of the C-4 hydroxyl of GalNAc (Scheme 3).

**Scheme 3:**
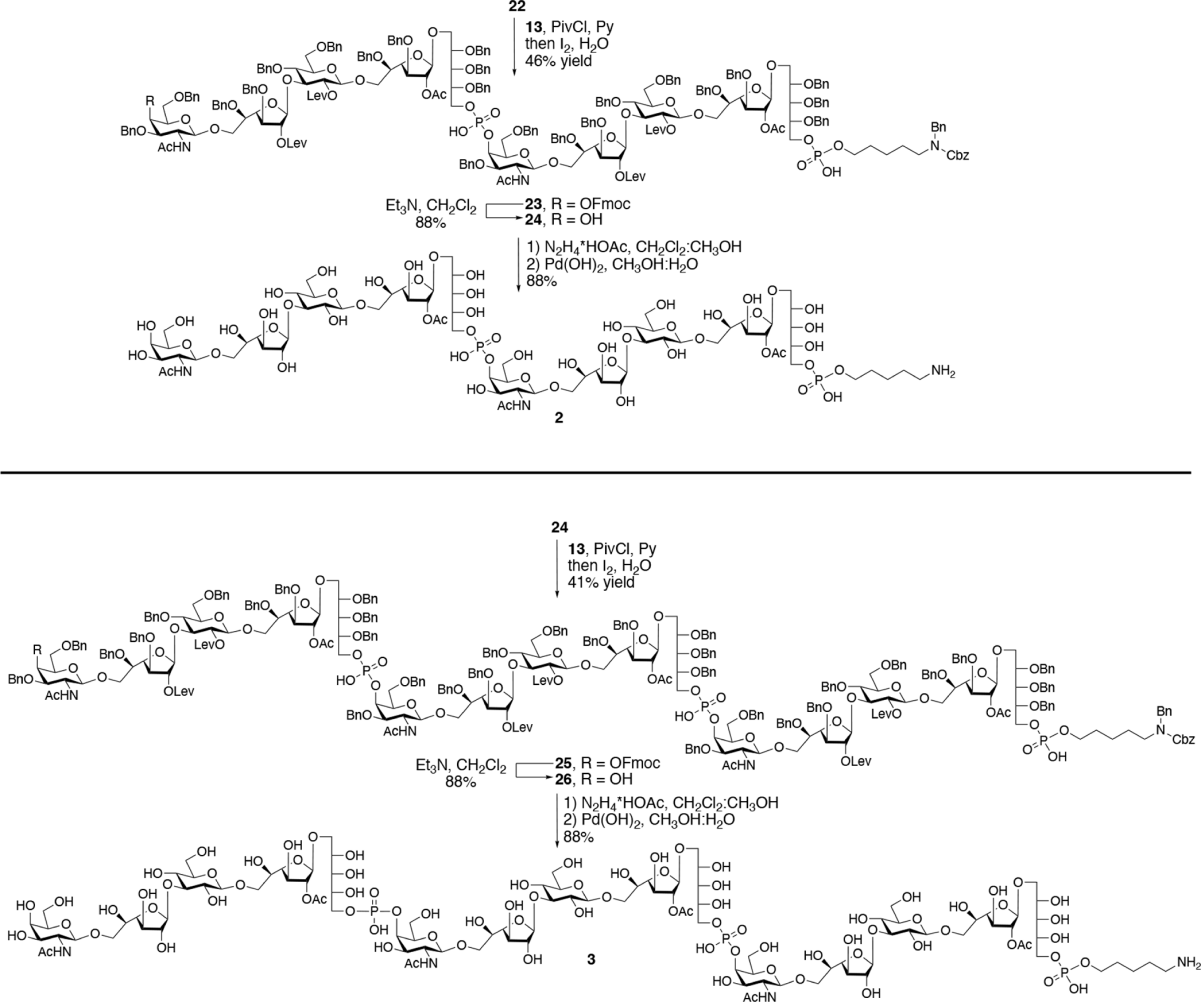
Synthesis of Decasaccharide **2** and Pentadecasaccharide **3**

### Microarray Development and Binding Studies with Ficolins and Factor Antisera

Next, attention was focused on developing a microarray to investigate the binding with ficolins and antisera. First, samples of compounds **1**–**3** were incubated with 100 mM NaOH (pH 11.0) at 40 °C for 2 h to affect de-*O*-acetylation to give compounds **4**–**6**. Next, the oligosaccharides were dissolved in a printing buffer (pH 8.5) and exposed to *N*-hydroxysuccinimide (NHS)-activated glass slides at a concentration of 100 μM in a replicate of 10. The acetyl esters were stable under these conditions.

The slides were incubated with different concentrations of recombinant l-ficolin having a C-terminal His-10 tag (2, 20, and 100 μg/mL) for 1 h, followed by washing and re-incubation with an AlexaFluor 647-conjugated anti-His antibody to detect binding. The results uncovered that the length of the CPS fragments together with *O*-acetylation is important for binding to ficolin-2 (Figure 3, left). Strong binding of l-ficolin was observed for *O*-acetylated trimer **3,** whereas little binding was detected for *O*-acetylated monomer **1** and a modest response was observed for *O*-acetylated dimer **2**. Removal of the acetyl esters abolished binding. Furthermore, compounds **1–6** exhibited no binding to ficolin-1 (M-ficolin). The data indicate that l-ficolin has complex binding requirements, which are dependent on not only *O*-acetylation but also the size of the ligand. A loss of *O*-acetylation of CPS is probably a mechanism of immune evasion, which has recently been reflected as a microevolution of serotype 35B into a genetically similar serotype 35D.^13^ The serotype 35D capsule is identical with serotype 35B except for the absence of acetyl esters at one of the galactofuranose residues. has a remarkable ability to adapt, and for example, there is an increase in the reported global occurrence of 35D infections in young children.^14^ A lack of *O*-acetylation of 35D CPS results in a loss of detection by l-ficolin, which in turn may explain its greater invasiveness.^26^

**Figure 3:**
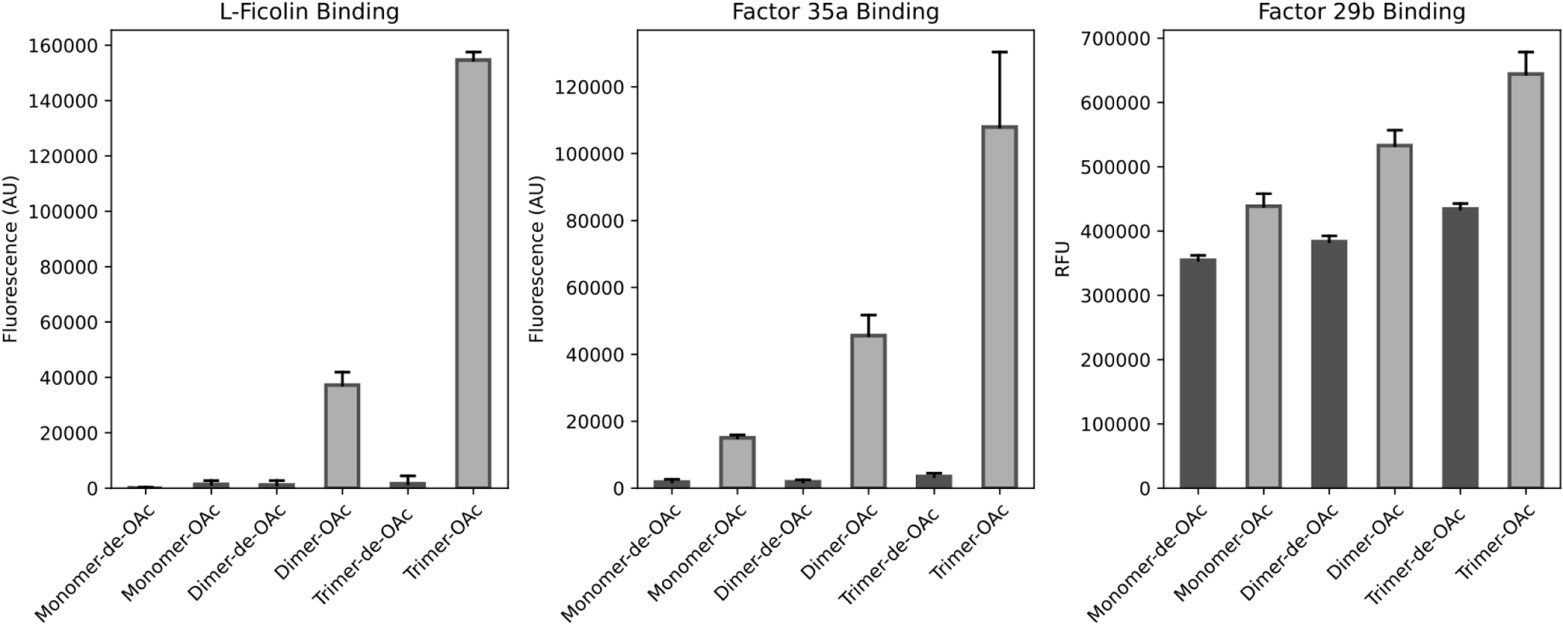
Results of microarray binding studies of synthetic oligosaccharides **1–6** with l-ficolin (left), factor 35a (middle), and factor 29b (right).

To further demonstrate the importance of previously neglected CPS *O*-acetylation, another set of microarray binding experiments was performed with serum factors 35a and 29b.^27^ Factor sera bind to specific serotypes and are used to perform detailed serotype identification. Factor serum 29b is reported to recognize both 35B and 35D, while factor serum 35a only recognizes type 35B.^13^ Thus, the slides were incubated with factor sera 29b and 35a at different dilutions, followed by washing and re-incubation with a premixed solution of biotinylated Goat-anti-Rabbit antibody and Streptavidin-AlexaFluor 647 conjugate for detection. The data showed that factor 35a only recognizes the *O*-acetylated oligosaccharides derived from the CPS of 35B, and thus, the acetyl esters are a critical part of the recognition epitope (Figure 3, middle). Length was also important for binding, and an increase in oligosaccharide length resulted in better responsiveness. Factor 29b bound to both acetylated 35B and the non-acetylated oligosaccharides (Figure 3, right), and in this case, the length of the oligosaccharides did not impact binding.

### Development of the l-Ficolin-Oligosaccharide Binding Model

A combination of solution-state NMR spectroscopy and computational simulation was employed to elucidate, at the molecular level, the binding interaction between *O*-acetylated compound **1** and l-ficolin. Initially, saturation transfer difference (STD) NMR experiments were performed to determine whether l-ficolin recognizes specific monosaccharide units of compound **1**. To further explore the bound-state conformation of compound **1**, we employed transfer ROE (trROE) experiments, which made it possible to calculate interproton distances in both the unbound and bound state. Any observed differences in interproton distances between the two states would indicate a conformational change in compound **1** upon binding to l-ficolin. This insight is not accessible through STD NMR. Finally, computational docking of the energy-minimized structure of **1** was performed to predict a plausible binding pose, which was compared with the NMR data. The docking model is in agreement with a previously published high-resolution X-ray crystal structure of the *N*-acetyl-choline–l-ficolin complex, corroborating the results obtained by NMR.

First, standard 2D NMR techniques, including COSY, HSQC, and HSQC-TOCSY, were used to assign the ^1^H and ^13^C resonances of **1** (see Pages S26–S32). These assignments enabled resonance identification in the later STD and ROE measurements. Next, we performed ^1^H STD NMR experiments to determine which monosaccharide units of **1** are in direct contact with the l-ficolin binding site (see Page S25). In STD NMR, selective saturation of a protein is performed, and the resulting magnetization is transferred to bound **1** through spin diffusion. Only protons of **1** that are in spatial proximity to the protein, typically within 5 Å, receive saturation and show a reduced signal in the STD difference spectrum. Therefore, the intensity of the STD signals of the protons of compound **1** reflects their relative proximity to the l-ficolin binding surface, enabling identification of the interacting monosaccharide partners. The resulting data clearly demonstrate binding of the acetyl (OAc) ester, while the *N*-acetyl (NHAc) moiety does not appear to be involved (Figure 4, bottom). Strong STD signals were also observed for all protons of the Galf2 residue and the ribitol moiety. In contrast, the absence of STD signals from the sugar ring protons of Glc, Galf4, and Gal*N* indicates that these monosaccharide residues do not make direct contact with l-ficolin. Altogether, the STD results point to the Galf2–ribitol portion of compound **1** as the primary site of interaction with l-ficolin.

**Figure 4:**
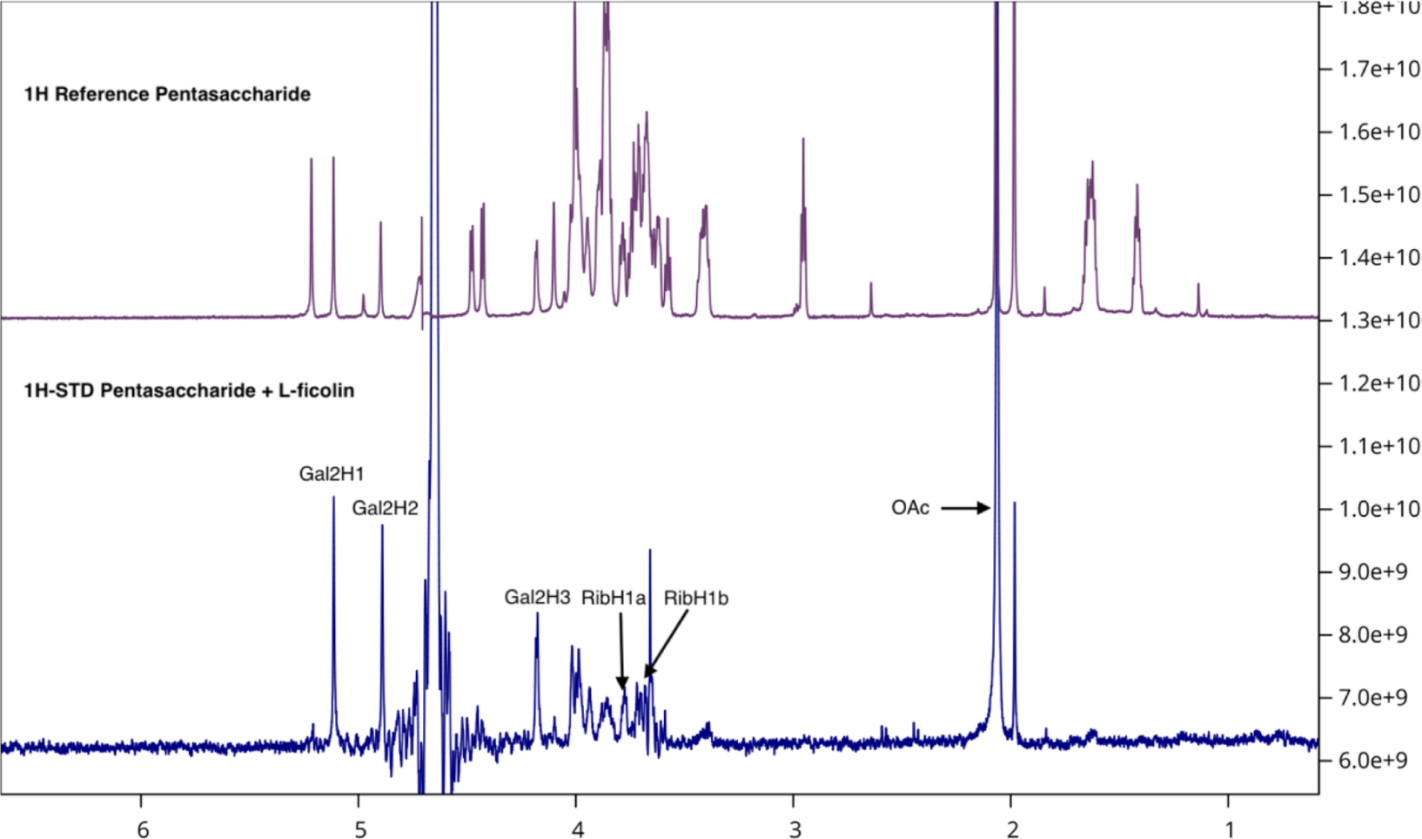
Top: reference ^1^H NMR spectrum of **1** (2.4 mM). Bottom: 1D 1H-STD NMR spectrum of **1** (2.4 mM) and 70 μM l-ficolin.

Subsequently, we employed ROESY and trROE experiments to examine whether binding to l-ficolin induces conformational changes in **1**. There are several advantages of using ROESY over NOESY in this study. First, across different field strengths and temperatures, ROE signal-to-noise ratios were consistently higher than those of NOEs. Second, ROESY required less experimental time while producing spectra with improved sensitivity. Optimal sensitivity was achieved when diagonal peak intensities decayed to approximately 2.7 times less than those at zero mixing time, i.e., 200 ms mixing time proved effective for our purposes. However, several challenges still had to be addressed. ROESY cross-peaks can be influenced by the offset dependence of spin-lock conditions, as well as by homonuclear Hartmann–Hahn transfer, which manifests as TOCSY-like artifacts.^28^ These occur when the effective spin-lock fields (including offset effects) for two coupled spins differ by less than ∼5×*J*_H–H_. In such cases, inaccurate distances may result for *J*-coupled protons separated by two or three bonds. To minimize these artifacts, we carefully optimized the experimental conditions and used a pulse sequence that relies on the “jump-symmetrized” adiabatic spinlock for mixing^29^ (see Page S25). The spin-lock pulse offset was positioned in the center of the spectrum (3.0 ppm), and a moderate spin-lock field strength was applied (272 μs = 3.6 kHz). Distance calculations were based on a single ROESY experiment with a 200 ms mixing time. This approach does not rely on initial rate approximations or external calibration.^30,31^ The interproton distances *r* were calculated^32^ from the cross-peak (*a*_*ij*_) and diagonal peak (*a*_*ii*_) intensities using eq[Disp-formula eq1] as follows:

1 $$r={\left(\frac{-0.2\tau_{c}\gamma^{4}\hbar^{2}{\left(\frac{\mu_{0}}{4\pi }\right)}^{2}}{\ln\left(\frac{a_{ii}d_{ii}+a_{ij}d_{ij}}{a_{ii}d_{ii}-a_{ij}d_{ij}}\right)}\left(\frac{6\tau_{c}}{1+4\omega_{0}^{2}}-\tau_{c}\right)\right)}^{\frac{1}{6}}$$

The correction factors,^33^ *d*_*ii*_ and *d*_*ij*_, are defined as follows:

2 $$d_{ii}=\frac{1}{{\left(\frac{\tan^{2}\theta_{i}}{1+\tan^{2}\theta_{i}}\right)}^{2}}$$

where

3 $$\tan\theta_{i}=\frac{\gamma B_{1}}{\omega_{i}-\omega_{0}}$$

Having interproton distances of unbound **1** determined (see Page S34), we proceeded with data collection for the l-ficolin −**1** complex. To this end, we employed the trROE, which is an attractive method described by Bax,^34^ Clore, and Gronenborn^35,36^ to unambiguously identify ligand protons that upon binding are in close proximity to the protein. This technique is particularly well-suited for studying oligosaccharide–protein interactions with a fast ligand dissociation rate (*k*_off_), as it allows for the efficient transfer of ROEs from the protein to the oligosaccharide via chemical exchange. Because of the rapid exchange between the bound and free states, the strongly enhanced ROEs of the bound state dominate over the much weaker and more slowly developing ROEs of the free oligosaccharide. As a result, trROEs provide direct information on the bound-state conformation of the oligosaccharide. Importantly, this type of conformational insight is not accessible through saturation transfer difference (STD) experiments alone, making trROE a complementary technique for probing the structural features of **1** when complexed with l-ficolin.

The modified ROESY pulse sequence^29^ did not require application of filters to suppress protein signals,^37^ as the ROESY mixing time is typically much longer than the protein T_1p_. Data were collected on an 800 MHz spectrometer by using a sample of **1** as a reference. Figure 5A shows the 200 ms ROESY spectrum of **1,** and Figure 5B shows the spectrum recorded in the presence of l-ficolin. The two spectra are qualitatively similar; however, quantitative analysis revealed notable differences upon binding (see Pages S37 and S38). Changes in inter-proton distances are plotted in Figure 5. Notably, in the l-ficolin-bound state, the distances associated with the Galf2 residue and the interglycosidic Galf2–Ribitol (Rib) pair are substantially increased.

**Figure 5:**
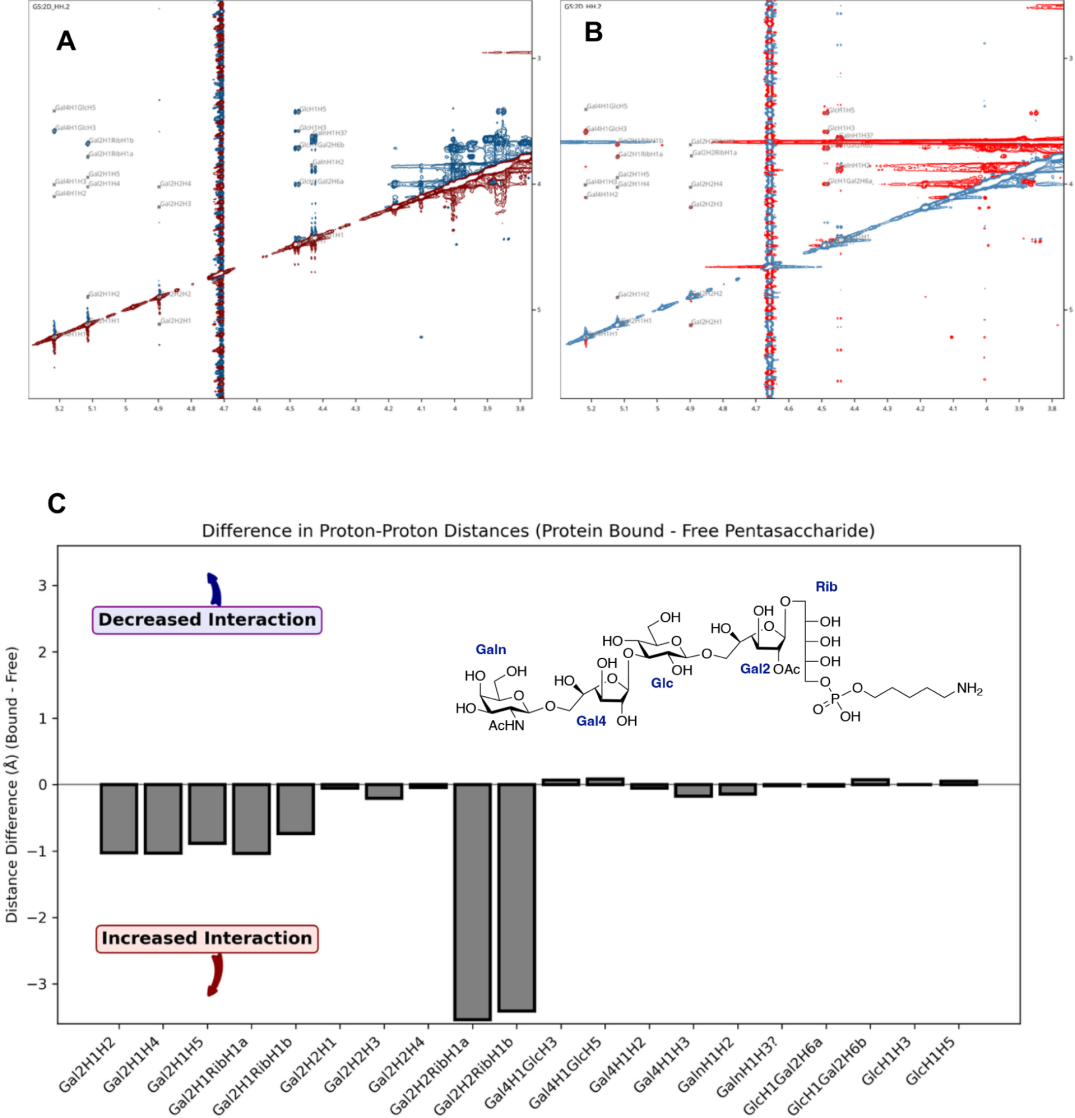
(A) 2 D ^1^H–^1^H ROESY spectrum of 2.4 mM **1**. (B) 2 D ^1^H–^1^H trROESY spectrum of 2.4 mM **1** with 70 μM l-ficolin. (C) Differences in proton–proton distances (**1** and **1** + l-ficolin).

The trROE results further support that the acetylated galactofuranoside (Galf2) and the ribitol moiety (Rib) of **1** interact directly with l-ficolin, as only their associated interproton distances were perturbed upon complex formation. As a matter of fact, all other interglycosidic distances, particularly those involving the anomeric protons of sugars, remained unchanged. Additionally, intra-ring proton distances within Galf2 became shorter, a change not observed in any other monosaccharide of compound **1**. This is indicative of Galf2 undergoing a conformational change.

To further support and structurally contextualize the NMR data, we performed computational docking experiments. To ensure comprehensive conformational sampling and to account for molecular flexibility, five independently seeded docking runs were carried out, generating a total of 100 binding poses. Figure 6A shows the top-ranked pose, corresponding to the lowest predicted binding energy (see Page S39). Structural analysis of the docked complex indicates that unrestrained molecular docking can qualitatively reproduce the binding mode between compound **1** and the putative l-ficolin binding site. In this pose, the ribitol moiety (Rib), the Galf2 residue, and the *O*-acetyl group are all directly involved in binding interactions, which is consistent with the STD and trROE NMR data. As illustrated in Figure 6B, the unbiased docking model also reproduces a network of interactions observed in the crystal structure of l-ficolin bound to acetylcholine,^38^ including hydrogen bonds and hydrophobic contacts with Asp133 and Arg132. The *O*-acetyl ester of Galf2 occupies the same binding pocket as the acetyl moiety of acetylcholine, supporting the notion that this interaction is a key driver for the recognition of *O*-acetylated compounds (Figure 6D).

**Figure 6:**
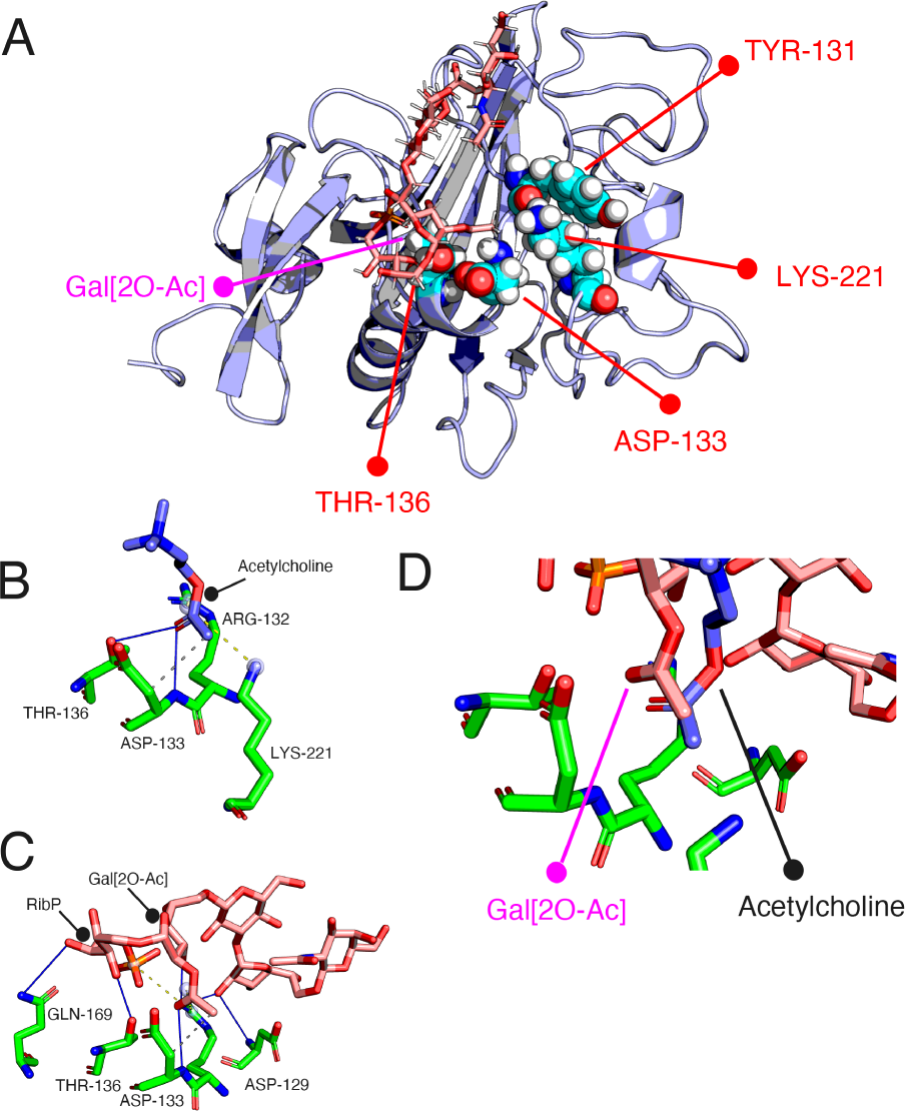
(A) Best-scoring docking model of the l-ficolin–**1** complex, highlighting key interactions between the Gal2 OAc moiety (magenta) and the putative binding site (red) for acetylated saccharides involving residues THR-136, ASP-136, LYS-221, and TYR-221. (B) X-ray crystal structure of *N*-acetylcholine in the presence of l-ficolin (PDB: 2J0H). (C) Close-up image of the l-ficolin–**1** complex. (D) Overlay of the X-ray crystal structure of *N*-acetylcholine and the docked l-ficolin–**1** complex.

## Conclusions

A scalable synthetic route for oligosaccharides composed of multiple repeating units derived from the CPS of 35B is described, which is an emerging serotype. Our modular synthetic approach provided compounds composed of up to 15 monosaccharides in length using a key pentasaccharide building block equipped with four orthogonal protecting groups. Careful selection of the protecting groups was important for preserving the biologically important *O*-acetyl esters and phosphodiester linkages. The synthetic compounds made it possible to develop a binding model of l-ficolin, which is one of the few proteins that can activate the lectin pathway of the complement system.^39^ There are indications that l-ficolin can recognize a diverse range of microbial *O*- and *N*-acetylated carbohydrate structures as well as polysaccharides such as 1,3-β-glucan.^11^ l-Ficolin-mediated complement deposition has been observed for serotypes 11A and 35B but not for variants deficient in the *O*-acetyltransferase.^12^ Furthermore, ficolin-a-deficient knockout mice exhibited reduced survival rates following infection with compared to e wild-type mice.^40^ Direct binding of l-ficolin to CPS of serotype 35B has not been demonstrated, and furthermore, molecular mechanisms by which l-ficolin distinguishes self from non-self are not well understood. Multiple binding sites have been identified within l-ficolin, and the S3 site, which includes Arg132, Asp133, Thr136, and Lys221, is the putative binding pocket for acetylated saccharides.^38^

We employed synthetic pentasaccharide **1**, which represents a single repeating unit of CPS 35B, and several NMR and computational techniques to unravel the molecular details of its binding to l-ficolin. Interproton distances of **1** were determined in the unbound and bound states by trROE experiments. These experiments established that the Galf­(OAc)-1,1-Ribitol moiety of **1** undergoes conformational changes upon binding to l-ficolin. STD NMR experiments confirmed that this disaccharide is the minimal motif of the serotype 35B capsular polysaccharide (CPS) required for binding. A binding pose obtained by computational docking agreed with the experimental data and revealed that the acetyl ester of **1** can be accommodated by the S3 site of l-ficolin, making key interactions with Arg132 and Asp133. It also showed that the galactofuranoside and ribitol moieties make direct interactions with the protein.

l-Ficolin is a pattern recognition receptor that can recognize a plethora of acetyl ester-containing oligosaccharides. The conformational flexibility of the furanoside^41^ makes it an attractive molecular recognition element for a pattern recognition receptor.

Binding experiments by microarray demonstrated that a compound composed of three repeating units (**3**) bound avidly to l-ficolin at a concentration that mimics its presence in serum. The binding was much stronger than that of a single repeating unit (**1**) even when presented on a surface. As expected, *O*-acetylation was critical for binding, and oligosaccharides derived from the CPS of serotype 35D, which is devoid of acetylation, were not recognized. Thus, a multivalent display of *O*-acetylated epitopes as part of a polysaccharide is probably important for detection by l-ficolin. This is in agreement with the observation that ficolins occur as oligomers due to a collagen-like region, and thus, it is likely that these immune receptors bind to saccharides that present multiple binding partners.^42^ Despite immune detection by l-ficolin, bacteria may have an advantage by *O*-acetylation of their capsular polysaccharides, and for example, loss of WciG-mediated *O*-acetylation of serotypes 33A and 33F resulted in a less stable capsule with an increase in cell wall accessibility, increased nonspecific opsonophagocytic killing, enhanced biofilm formation, and increased adhesion to nasopharyngeal cells.^43^

Binding studies with antisera indicated that the acetyl ester of the d-galactofuranose moiety of 35B CPS is critical for binding and appears to be an immune-dominant feature. The results indicate that immunization with 35B CPS will not provide protection against 35D, and thus, inclusion of the 35B CPS serotype in a vaccine may result in serotype replacement by 35D. Interestingly, antibodies that can bind 35D can also recognize 35B, and thus, 35D CPS may provide cross-protection, but this needs further investigation.

It is a challenge to preserve acetyl esters during the industrial-scale production of conjugate vaccines. The synthetic antigens described here are modified by an aminopentyl linker that will facilitate controlled coupling to carrier proteins such as CRM_197_ and conjugate vaccine development. The synthetic strategy described here is also relevant to the preparation of other emerging serotypes of that share structural features, such as repeating units linked by phosphodiester bonds and acetyl esters.

## Supplementary Material



## Notes

Methods, analytical data, and additional figures (PDF)
